# The connection of motor improvement after deep brain stimulation in Parkinson’s disease and microstructural integrity of the substantia nigra and subthalamic nucleus

**DOI:** 10.1016/j.nicl.2024.103607

**Published:** 2024-04-18

**Authors:** Marco G. Hermann, Nils Schröter, Alexander Rau, Marco Reisert, Nadja Jarc, Michel Rijntjes, Jonas A. Hosp, Peter C. Reinacher, Wolfgang H. Jost, Horst Urbach, Cornelius Weiller, Volker A. Coenen, Bastian E.A. Sajonz

**Affiliations:** a Department of Stereotactic and Functional Neurosurgery, Medical Center – University of Freiburg, Faculty of Medicine, University of Freiburg, Freiburg, Germany; b Department of Neurology and Clinical Neuroscience, Medical Center – University of Freiburg, Faculty of Medicine, University of Freiburg, Freiburg, Germany; c Department of Neuroradiology, Medical Center – University of Freiburg, Faculty of Medicine, University of Freiburg, Freiburg, Germany; d Department of Diagnostic and Interventional Radiology, Medical Center – University of Freiburg, Faculty of Medicine, University of Freiburg, Freiburg, Germany; e Medical Physics, Department of Radiology, Medical Center – University of Freiburg, Faculty of Medicine, University of Freiburg, Germany; f Fraunhofer Institute for Laser Technology (ILT), Aachen, Germany; g Parkinson-Klinik Ortenau, Wolfach, Germany; h Center for Deep Brain Stimulation, University of Freiburg, Germany

**Keywords:** Parkinson’s disease, Deep Brain Stimulation, Therapy response, Diffusion Microstructure Imaging (DMI), Microstructural integrity

## Abstract

**Background:**

Nigrostriatal microstructural integrity has been suggested as a biomarker for levodopa response in Parkinson’s disease (PD), which is a strong predictor for motor response to deep brain stimulation (DBS) of the subthalamic nucleus (STN). This study aimed to explore the impact of microstructural integrity of the substantia nigra (SN), STN, and putamen on motor response to STN-DBS using diffusion microstructure imaging.

**Methods:**

Data was collected from 23 PD patients (mean age 63 ± 7, 6 females) who underwent STN-DBS, had preoperative 3 T diffusion magnetic resonance imaging including multishell diffusion-weighted MRI with b-values of 1000 and 2000 s/mm^2^ and records of motor improvement available.

**Results:**

The association between a poorer DBS-response and increased free interstitial fluid showed notable effect sizes (rho > |0.4|) in SN and STN, but not in putamen. However, this did not reach significance after Bonferroni correction and controlling for sex and age.

**Conclusion:**

Microstructural integrity of SN and STN are potential biomarkers for the prediction of therapy efficacy following STN-DBS, but further studies are required to confirm these associations.

## Highlights

• Regions of Interst‘s Integrity is quantified with Diffusion Microstructure Imaging.
• More free interstitial fluid in SN and STN is linked to poorer response to STN-DBS.
• Microstructural integrity is a potential biomarker for therapy efficacy of STN-DBS.

## 1 Introduction

Parkinson's disease (PD) is a progressive neurodegenerative disorder characterized by the degeneration of dopaminergic neurons in the substantia nigra (SN), leading to typical motor symptoms such as akinesia, rigidity, postural instability and tremor (Greffard et al., 2006).

After an initial good response to dopaminergic medication, as the disease progresses, levodopa-induced complications such as motor fluctuations can occur (Bloem et al., 2021).

Deep brain stimulation (DBS) targeting the subthalamic nucleus (STN) is an established and effective treatment for patients with PD and motor fluctuations or therapy-resistant tremor (Deuschl et al., 2006, Schuepbach, 2013). While the precise mechanism of action remains elusive, STN-DBS is believed to diminish pathologically increased activity in the STN, leading to a reduction in the cardinal symptoms of PD (Krack et al., 1998, Limousin et al., 1998).

To maximize individual benefits for patients and minimize the risk of adverse side effects, strict criteria are essential for selecting patients for surgery. Apart from a younger age, no or very mild cognitive impairment, absence or well-controlled psychiatric disease, and a minimum of psychosocial independence and/or support, patients should demonstrate an excellent response to levodopa as it is a strong predictor for response of motor symptoms to DBS (Hariz and Blomstedt, 2022, Lin et al., 2022).

The levodopa challenge test (LCT) measures the motor response to a suprathreshold dose of levodopa by comparing scores on the Movement Disorder Society Unified Parkinson’s Disease Rating Scale Part III (MDS-UPDRS-III) in the “defined-ON condition” (best therapeutic effect after medication agreed by patient and physician) with those in the “defined-OFF condition” (at least 12 h after receiving the last levodopa medication dose and after withdrawal from dopamine agonists) (França et al., 2022, Saranza and Lang, 2021). Motor response in the LCT can predict the motor outcome after STN-DBS both qualitatively and quantitatively (Lachenmayer et al., 2021), probably by indirectly reflecting the functional integrity of structures outside the presynaptic nigrostriatal dopaminergic pathway (França et al., 2022, Jergas et al., 2022).

However, the drawbacks of the LCT include significant discomfort resulting from discontinuing dopaminergic medication and various factors, both investigator- and patient-dependent, leading to a certain degree of subjectivity of the LCT results. Furthermore, patients who narrowly miss the 30 % response target in LCT pose a challenge in terms of qualifying for DBS, and an additional reliable biomarker is desirable in this context.

To objectively measure the integrity of the brain, various MRI-based imaging techniques can be employed. Here, advanced approaches such as Diffusion Microstructure Imaging (DMI) allow for the non-invasive approximation of the brain’s microstructure (Reisert et al., 2017). For this, DMI relies on the “standard model” (Novikov et al., 2019, Reisert et al., 2017, Zhang et al., 2012) to disentangle microstructural compartments comprising the intra-axonal fraction (V-intra, including dendrites and myelinated axons), an extra-axonal fraction (V-extra, consisting of neuronal somata and unmyelinated axons), and a free fluid fraction (V-CSF).

Microstructural integrity of the SN and putamen, measured with DMI, was shown to be a promising biomarker not only for motor impairment but also for levodopa response in patients with PD (Schröter et al., 2022). Hence, this technique might be predictive for response to STN-DBS, as well.

We thus employed DMI to investigate the association of microstructural degeneration of SN, STN, and putamen with motor response to STN-DBS.

## 2 Methods

### 2.1 Participants

We report data from patients who had given informed consent to participate in our prospective DBS registry (trial registration number: DRKS00025490) and were admitted for DBS surgery to the Department of Stereotactic and Functional Neurosurgery, Medical Center–University of Freiburg between 06/17/2020 and 11/17/2021. Inclusion criteria for this study were

(1.) available preoperative 3 T MRI, including artifact-free multishell diffusion MRI, (2.) STN-DBS implantation in our hospital as exemplarily reported before (Reinacher et al., 2019), recommended by our interdisciplinary movement disorder conference due to clinically established PD fulfilling the consensus guideline criteria (Postuma et al., 2015), (3.) a maximum of 1 year between preoperative clinical assessment and DBS surgery, and (4.) discontinuation of dopaminergic medication with adequate latency before Med OFF test of motor performance (see below).

The study follows the tenets of the declaration of Helsinki and was approved by the local ethics committee (21-1274).

### 2.2 Clinical testing

Motor impairment and DBS-associated motor improvement were assessed with the MDS-UPDRS-III (Goetz et al., 2008) at the following time points: a) preoperatively after discontinuation of dopaminergic medication for at least 12 h in the Med OFF-state, b) at follow up closest to 12 months postoperatively in Stim ON Med OFF state after discontinuation of dopaminergic medication for at least 10 h. DBS-response was calculated as improvement in MDS-UPDRS-III between the above mentioned conditions in percent. UPDRS-III was applied for preoperative testing in 5 patients whose scores were calibrated to MDS-UPDRS-III values according to Goetz et al. (2012) prior to further analyses.

### 2.3 Imaging acquisition and analysis

MRI acquisition, normalization and calculation of DMI parameters were performed as previously described (Schröter et al., 2022). In brief, preoperative 3 T MRIs (MAGNETOM Prisma, Siemens Healthcare, Erlangen, Germany) including a multishell dMRI sequence with b-values of 1000 and 2000 s/mm^2^ were transferred to a local instance of the postprocessing platform NORA (www.nora-imaging.org) for further analysis. Following pre-processing of the diffusion-weighted images, we estimated microstructural diffusion metrics based on a three-compartment diffusion model using a Bayesian approach (Reisert et al., 2017, https://bitbucket.org/reisert/baydiff/src/master/). We determined (I) the free water/CSF fraction (V-CSF), (II) the volume fraction within neuronal processes (V-intra) and (III) the volume fraction outside the neuronal processes (V-extra), each corresponding to the above-mentioned structures (Schröter et al., 2022). T1w-imaging datasets were segmented into white matter, gray matter and cerebrospinal fluid (CSF) using CAT12 (https://www.neuro.uni-jena.de/cat/). DMI images were co-registered to the T1w images. The validity of co-registration between DMI images, T1w and binary masks was manually confirmed. Quality control involved visually inspecting each individual DMI dataset and CAT12 segmentation.

We extracted DMI parameters for the SN, STN and putamen using an atlas-based approach (Ilinsky et al., 2018, Rolls et al., 2020).

Electrode locations and volumes of activated tissue (VAT) were generated based on postoperative CT with Brainlab Elements (Brainlab, Munich, Germany) and coregistered to Montreal Neurological Institute space on the NORA imaging platform. The Euclidean distance (mm) to reported sweet spots (Akram et al., 2017, Bot et al., 2018, Caire et al., 2013, Dembek et al., 2019, Horn et al., 2017) from the center of both VATs was calculated for each patient.

### 2.4 Statistical analyses

Statistical Analyses were performed using R (version 4.1.0, https://www.R-project.org/) and GraphPad Prism 9.5.1 (GraphPad Software, San Diego, CA, USA). Shapiro-Wilk test was used to assess normal distribution of data.

Corresponding to our previous study on the correlation of clinical outcome parameters with DMI parameters, we primarily focused on the free water-/CSF-fraction (V-CSF) as a correlate of cellular demise or microstructural disintegration. Hence, we examined associations between V-CSF values from the SN, STN and putamen contralateral to the clinically more affected side and motor response to STN-DBS with partial correlation analyses controlling for age and sex (Kim, 2015). We did not additionally correct for Euclidean distances to sweet spots (right, left, mean of both sides and contralateral to clinically more affected side), as explorative Pearson correlation analyses did not reveal any significant associations with DBS response. The α-level for the confirmatory analyses was adjusted with the Bonferroni method correcting for multiple comparisons. P −values ≤ 0.016 were considered statistically significant.

Additional exploratory analyses were conducted to illustrate (1) the association between microstructural alterations in the SN, STN, and putamen via Pearson’s correlation coefficient, (2) the generally expected effect of STN-DBS on motor improvement and LED reduction using two-tailed paired t-tests, (3) the association between levodopa responsiveness in LCT and DBS response employing Pearson correlation analysis, (4) the association between V-CSF of the STN contralateral to the clinically more affected side and baseline preoperative motor performance in the medication OFF state via partial correlation.

## 3 Results

### 3.1 Participants

Inclusion criteria were met by 23 patients who received DBS implantation between 06/17/2020 and 11/17/2021. Demographic and clinical data of the included patients are provided in Table 1.

**Table 1:** Demographics and Clinical Characteristics.

**Parameter**	**n**
n	23
Sex (Male/Female)	17/6
Clinically more affected side (Right:Left)	11:12
	**mean ± SD (range)**
Age at surgery (years)	63 ± 7 (50–73)
Disease Duration at surgery (years)	10.57 ± 4.48 (5–21)
Total Levodopa Equivalent Dose preOP (mg)	1285 ± 373 (525–2043)
Dopamine agonist portion of the Levodopa Equivalent Dose preOP (mg)	251 ± 168 (0–780)
Hoehn & Yahr Stage preOP Med OFF	3 ± 1.02 (2–5)
Hoehn & Yahr Stage preOP Med ON	2.26 ± 0.54 (2–4)
MDS-UPDRS-III preOP Med OFF	53.18 ± 21.04 (22.6–111)
% Levodopa Responsiveness in LCT preOP	54.88 ± 17.39 (27.37–92)*
Time between preOP MDS-UPDRS-III and surgery (days)	26.57 ± 51.89 (0–238)
Time since DBS implantation at FU_12M_ (months)	13.3 ± 2.12 (9–17)
Total Levodopa Equivalent Dose FU_12M_ (mg)	499 ± 328 (75–1375)
LED reduction after DBS in %	62 ± 21 (9–95)
Dopamine agonist portion of the Levodopa Equivalent Dose FU_12M_ (mg)	74 ± 75 (0–240)
MDS-UPDRS-III FU_12M_ Stim ON Med OFF	28.17 ± 13.95 (9–63)
DBS response in %	43.71 ± 25.7 (−12.82–83.02)

Abbreviations:

DBS, Deep Brain Stimulation; FU_12M_; follow up closest to 12 months after surgery; LCT, levodopa challenge test; MDS-UPDRS-III, Movement Disorder Society Unified Parkinson’s Disease Rating Scale Part III; Med OFF, medication OFF state; Med ON, medication ON state; preOP, preoperatively; SD, standard deviation; Stim ON, stimulation ON state.

* A single patient with < 30 % levodopa responsiveness in the formal preoperative LCT showed better motor performance at discharge in best medical ON condition compared to the OFF condition (>30 %) and was hence considered a good candidate for STN DBS.

### 3.2 Predictors of STN-DBS success

**Fig. 1:**
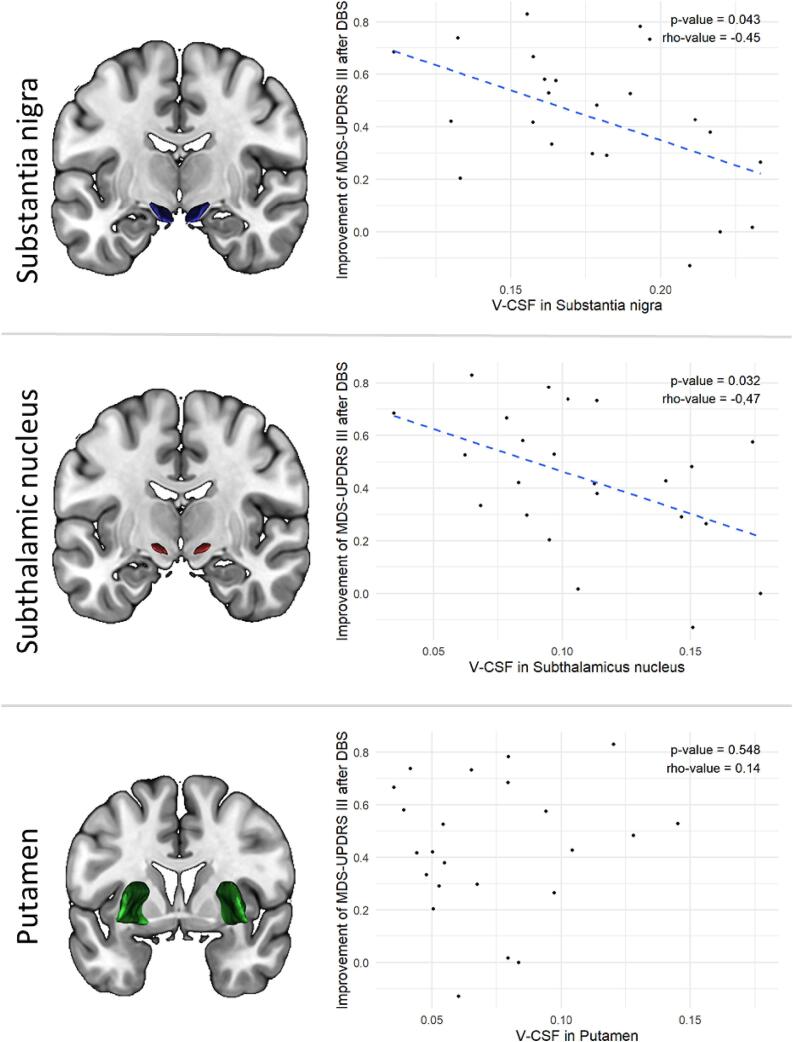
Association of microstructural free fluid (V-CSF) in Putamen, Substantia nigra and subthalamic nucleus with motor improvement by STN-DBS controlling for age and sex. Each dot represents a single patient. Dashed lines are used to point out the bigger effect sizes of the associations in the two upper graphs, which however do not reach the α-level of 0.016.

We observed negative associations with notable effect sizes between the improvement in MDS-UPDRS-III after DBS and the amount of free fluid (V-CSF) in SN (rho = -0.45, p = 0.043) and in the STN (rho = -0.47, p = 0.032), however, without statistical significance after Bonferroni correction of the α-level (Fig. 1). In contrast, a small effect size was observed upon testing the association between motor improvement and V-CSF in the putamen (rho = 0.14, p = 0.548).

To assess the quality of the DBS electrode implantation itself, we tested the distances (right, left, mean of both sides and contralateral to clinically more affected side) from the center of both VATs to the aforementioned sweet spots for an association with the DBS response. Here, we did not note a statistically significant association (all p > 0.05). Descriptive values of the stimulation parameters and the Euclidean distances between the center of the VAT and the sweet spots are provided in Supplementary Table 1 and 2.

### 3.3 Exploratory analyses

All data was normally distributed except for the following two parameters: LED at FU_12M_, Euclidean distance from the center of the left VAT to the left sweet spot of Horn/Caire (Caire et al., 2013).

Microstructural alterations in the SN and STN were significantly associated (r = 0.583, p = 0.01), while no association of the putamen with STN (r = 0.258, p = 0.34) or SN (r = 0.211, p = 0.34) was found.

As expected, STN-DBS resulted in significant motor improvement (t (22) = 5.698, p < 0.0001) and reduction of LED (t (22) = 11.27, p < 0.0001) across the group (Table 1).

We did not find an association between levodopa responsiveness in LCT and DBS response (rho = -0.09, p = 0.70). Additionally, partial correlation could not confirm an association between microstructural integrity of the STN and preoperative motor performance in the Med OFF condition whether corrected for age and sex (p = 0.956) or not (p = 0.656).

## 4 Discussion

We investigated the basal ganglia microstructure in a prospective sample of patients with PD who received STN-DBS. Though not reaching statistical significance, the observed decent effect size of the association between motor improvement by STN-DBS and the microstructural integrity of the SN and STN indicates a potential connection. In contrast, only a small correlation was found for the putaminal microstructure or motor improvement in preoperative LCT. The negative correlation coefficients of V-CSF (as a surrogate for disintegration) and DBS response point to the requirement of microstructural integrity of the SN and STN.

### 4.1 Microstructural integrity in regions of interest

We observed a potential negative connection between the degree of nigral degeneration and the response to STN-DBS. This is in line with the most popular theory on the mode of action of STN-DBS disrupting pathological hyperactivity along the hyperdirect and indirect basal ganglia pathway and thereby allowing for more prokinetic output based on SN activity (cf. Chiken and Nambu, 2016).

The analysis of STN microstructure indicated a varying distribution of free fluid fraction within our sample, implying gradual differences in the composition of the STN. This could be either due to the loss of cells or to the loss of axons and dendrites as both lead to an increase in the free fluid fraction and thus more pronounced neurodegeneration which in turn was associated with a poorer response to DBS. Functionally, (larger) STN lesions are expected to result in a reduction of PD motor symptoms (Aziz et al., 1991, Benazzouz et al., 2002, Bergman et al., 1990, Martínez-Fernández et al., 2023, Martínez-Fernández et al., 2020, Martínez-Fernández et al., 2018). Exploratory analyses, however, could not confirm a relationship between baseline motor performance and microstructural integrity of the STN in our sample which could also be due to the small sample size. Previous histopathological (Hardman et al., 1997, Mazumder et al., 2022) and microstructural MRI studies (Burciu et al., 2017, Mitchell et al., 2019) observed no degeneration of the STN measurable by these methods in the course of PD. Animal models (both rodent and primate) showed a reduction in the number of cortico-STN synaptic terminals and synaptic connection strength (Chu et al., 2017, Mathai et al., 2015, Wang et al., 2018) which could explain the detectable increase of V-CSF.

The fact that we did not observe a relevant strength of association of DBS response with the putamen can be explained by the way patients’ eligibility for DBS is evaluated: In light of the prerequisite for a sustained response to levodopa as a fundamental criterion for STN-DBS, coupled with the necessity for well-preserved putaminal integrity (Schröter et al., 2022), a patient selection process emerges that favors individuals who exhibit minimal degeneration within their putamen. Consequently, this results in reduced variability within the patient population regarding putaminal integrity and levodopa response. In line with this, we encountered a rather small variance in DMI metrics in the putamen compared to both SN and STN.

In general, precise positioning of the DBS electrode is crucial for motor improvement. In this study, we did note small Euclidean distances with overall low variance between the center of VAT and stimulation sweet spots (Akram et al., 2017, Bot et al., 2018, Caire et al., 2013, Dembek et al., 2019, Horn et al., 2017). This well explains that we only noted small correlation coefficients between these distances and DBS response. Moreover, the validity of the impact of microstructural integrity of target regions on DBS response is thereby corroborated by the high uniformity of electrode positioning.

### 4.2 Potential role of diffusion microstructure imaging as a biomarker

Given that progressive SN degeneration is the major pathological process in PD (Spillantini et al., 1997), our results point to the aspect that DBS surgery might be more effective in improving motor symptoms in suitable patients at an earlier point of the microstructural demise of SN and STN. STN-DBS has been shown to improve motor symptoms in both younger and older patients (de Noordhout et al., 2022, Hariz and Blomstedt, 2022, Schuepbach, 2013, Shalash et al., 2014) and the current state of research regarding a direct association between younger age or shorter disease duration and motor improvement (Muellner et al., 2016) as well as quality of life (Geraedts et al., 2020) is inconclusive. These heterogeneous findings indicate that additional biomarkers are needed to better estimate patient’s suitability for STN-DBS. Our results present microstructural integrity of SN and STN as an intriguing potential biomarker in this regard deserving further scientific exploration.

Estimating cortical thickness with anatomical 3D T1-images, Muthuraman et al. (2017) revealed that the integrity of the frontal cortex (namely the paracentral area as well as the superior frontal region) can predict the effects of STN-DBS in patients with PD. Although this association also requires further investigation in future studies, it could act synergistically with the analysis of STN and SN microstructure (using DMI) as a predictor for the clinical outcome of STN-DBS.

Apart from STN-DBS, another target for DBS in patients with PD mainly experiencing motor fluctuations and/or dyskinesias is the globus pallidus internus (GPi). Despite being overshadowed by STN-DBS in most countries nowadays (Hariz and Blomstedt, 2022), the GPi offers several advantages, including a direct anti-dyskinetic effect, easier programmability in outpatient settings, greater flexibility in medication adjustments and lenience for patients with advanced PD (Au et al., 2021, Hariz and Blomstedt, 2022). Deciding between STN- and GPi-DBS is influenced by differences in clinical effects, side effects, complications, programming, economic aspects, and other factors (Ramirez-Zamora and Ostrem, 2018, Williams et al., 2014, Xu et al., 2017). This decision therefore requires a patient-specific and interdisciplinary evaluation (Ramirez-Zamora and Ostrem, 2018, Williams et al., 2014). The aim is to tailor the therapy decision to the individual symptoms, characteristics, and expectations of the patient (Au et al., 2021, Xu et al., 2017). As in STN-DBS, a good response in the LCT predicts motor response to GPi-DBS, too (Lin et al., 2022). Microstructural integrity of the GPi measured with DMI might thereby provide an objective indicator to facilitate clinical decision-making. Specifically patients with preserved GPi integrity and a good response in the LCT, coupled with reduced microstructural integrity of the STN, might benefit more from GPi-DBS in terms of motor response. Future DMI studies focusing on the GPi could hence be of interest.

The integrity of SN and STN could also serve as valuable indicators for patients who fall into the gray area regarding their levodopa response. Therefore, a prospective study should investigate whether patients who fail to meet the 30 % response cutoff criterion for STN-DBS but still have intact microstructure of the SN and STN might nevertheless benefit from STN-DBS.

Furthermore, the assessment of SN and STN integrity allows us to gain insight into long-term effectiveness since the response was determined in a 1-year follow-up. This is highly relevant not only for the treating neurologist but also for the patients themselves, as it could be supportive in planning for the subsequent years.

### 4.3 Limitations

Though the enrolled sample size is rather small, these preliminary findings identify SN and STN microstructure as potential biomarker in STN-DBS. Confirmation of the results in larger samples, preferably across multiple centers is warranted. Here the proposed DMI approach itself is applicable to a multisite setting as it provides robust parameter estimation based on multishell dMRI data with rather short scanning time (Kellner et al., 2022, Reisert et al., 2017, Schröter et al., 2022).

We did not find a correlation between preoperative levodopa-responsiveness in the LCT and motor improvement following STN-DBS. Although the predictive value of the preoperative levodopa-responsiveness has been demonstrated in reviews and *meta*-analyses (Lachenmayer et al., 2021, Lin et al., 2022), this correlation is not constantly found across studies (Fasano et al., 2010, Piboolnurak et al., 2007, Tsai et al., 2009, Zaidel et al., 2010). Hence, levodopa responsiveness is useful to exclude non-responders to levodopa, but it is insufficient to predict DBS-efficacy especially on an individual level (Wolke et al., 2023). Thus, establishing other, paraclinical biomarkers such as neuroimaging is desirable (Lin et al., 2022, Wolke et al., 2023). Furthermore, the limited sample size may have compromised the statistical power necessary to identify a significant effect for levodopa responsiveness. This is corroborated by the fact that statistically significant associations were primarily found in studies with larger sample sizes or in *meta*-analyses/systematic reviews (Lachenmayer et al., 2021, Lin et al., 2022).

While all dopaminergic medications were discontinued at least 10 h (10 h in one patient, ≥12 h in the rest of the patients) prior to the motor examination at the FU_12M_ assessment, dopaminergic agonists sometimes have effects beyond this timeframe (Brooks, 2000). In addition, long-lasting compensatory mechanisms persisting even after overnight withdrawal from levodopa can lead to a 30 % decrease in MDS-UPDRS-III scores compared to dopamine-naïve OFF scores (Cilia et al., 2020). Consequently, the motor condition may have been better than the true OFF-state at FU_12M_ in some patients, potentially leading to an overestimation of the motor improvement due to DBS.

On the other hand, examinations at FU_12M_ in Med OFF Stim ON (with regular stimulation parameters, i.e. without a compensatory increase to mitigate the levodopa-withdrawal) underestimate the true potential for DBS response. This might counterbalance the medication-related limitations discussed before.

Clinical preoperative data were derived retrospectively from clinical routine testing. However, by using the widely recognized, highly standardized MDS-UPDRS-III test (Goetz et al., 2008) the interrater variability can be considered small. Furthermore, all raters were highly experienced movement disorder specialists at a tertiary referral center trained in MDS-UPDRS-III.

The diagnostic accuracy for PD in a clinical setting is approximately 80 % (Adler et al., 2014, Rizzo et al., 2016), and post-mortem validated diagnoses were not available for the enrolled cohort. Consequently, there may be patients included who had conditions other than PD.

However, only patients with clinically established PD and a long-term disease course as well as clinical follow-ups without red flags pointing to diseases other than PD were included after careful preoperative evaluation in an interdisciplinary movement disorders conference. All of this supports the diagnosis of PD in the patients within our cohort. Moreover, 16 out of 23 patients underwent [^18^F]fluorodeoxyglucose positron emission tomography as an additional diagnostic procedure, ensuring increased diagnostic certainty (Meyer et al., 2017).

## 5 Conclusion

Our findings suggest that microstructural integrity of the SN and STN influence motor outcome following STN-DBS in PD patients. Larger studies are required to further disentangle the particular roles of microstructural integrity of SN and STN and establish their value as a biomarker to estimate motor response to DBS and to help evaluate the suitability of patients for STN-DBS surgery preoperatively.

## 6 Financial disclosures for all authors (preceding 12 months)

**NS** received honoraria from Abbvie (presentation), STADAPHARM (advisor), Novartis (presentation). **AR** was supported by Berta-Ottenstein-Programme for Clinician Scientists, Faculty of Medicine, University of Freiburg. **JAH** was supported by Berta-Ottenstein-Programme for Advanced Clinician Scientists, Faculty of Medicine, University of Freiburg. **PCR** receives research support from: Else Kröner-Fresenius Foundation (Germany) and Fraunhofer Foundation (Germany). He is a consultant for Boston Scientific (USA), Inomed (Germany) and Brainlab (Germany) and has received honoraria for lectures from Arkana, Germany. **WHJ** received honoraria from Abbvie, Bial, Desitin, Zambon. **HU** received honoraria for lectures from Biogen, Eisai, Mbits and Lilly, is supported by German Federal Ministry of Education and Research, and is coeditor of Clin Neuroradiol. **VAC** receives a collaborative grant from BrainLab (Munich, Germany). He serves as an advisor for Aleva (Lausanne, Switzerland), Ceregate (Hamburg, Germany), Cortec (Freiburg, Germany) and Inbrain (Barcelona, Spain). He has an ongoing IIT with Boston Scientific (USA). He has received travel support and honoraria for lectures from Boston Scientific (USA), UNEEG Medical (Munich, Germany) and Precisis (Heidelberg, Germany). **BEAS** receives a research grant from Ceregate (Hamburg, Germany) and honoraria as an advisor for Precisis (Heidelberg, Germany). **MGH, MRe, NJ, MRi and CW** have nothing to report.

## CRediT authorship contribution statement

**Marco G. Hermann:** Writing – original draft, Software, Validation, Formal Analysis, Investigation, Data Curation. **Nils Schröter:** Writing – original draft, Validation, Methodology, Formal analysis, Data curation, Conceptualization. **Alexander Rau:** Writing – original draft, Visualization, Validation, Methodology, Formal analysis, Conceptualization. **Marco Reisert:** Writing – review & editing, Software. **Nadja Jarc:** Writing – review & editing, Investigation. **Michel Rijntjes:** Writing – review & editing, Resources. **Jonas A. Hosp:** Writing – review & editing. **Peter C. Reinacher:** Writing – review & editing. **Wolfgang H. Jost:** Writing – review & editing, Resources. **Horst Urbach:** Writing – review & editing, Resources. **Cornelius Weiller:** Writing – review & editing, Resources. **Volker A. Coenen:** Writing – review & editing, Resources. **Bastian E.A. Sajonz:** Writing – original draft, Conceptualization, Methodology, Validation, Formal analysis, Investigation, Data curation, Supervision, Project administration.

## Declaration of Competing Interest

The authors declare that they have no known competing financial interests or personal relationships that could have appeared to influence the work reported in this paper.

## Data Availability

Data will be made available on request.
