# Supplementary material for: The connection of motor improvement after deep brain stimulation in Parkinson’s disease and microstructural integrity of the substantia nigra and subthalamic nucleus
Source: Neuroimage Clin. 2024 Apr 18;42:103607. doi: 10.1016/j.nicl.2024.103607 (PMC11046219; doi:10.1016/j.nicl.2024.103607)
Supplement: Supplementary Data 1 [file mmc1.pdf]

The connection of motor improvement after deep brain stimulation in Parkinson's disease and microstructural integrity of the substantia nigra and subthalamic nucleus

Marco G. Hermann[1§]; Nils Schröter, MD[2,§,] Alexander Rau, MD[3,4,§]; Marco Reisert, PhD[1,5]; Nadja Jarc, MD[1], Michel Rijntjes, MD[2]; Jonas A. Hosp, MD[2]; Peter C. Reinacher, MD[1,6], Wolfgang H. Jost, MD[7]; Horst Urbach, MD[3]; Cornelius Weiller, MD[2]; Volker A. Coenen, MD[1,8], Bastian E. A. Sajonz, MD, MSc[1]

[1] Department of Stereotactic and Functional Neurosurgery, Medical Center – University of Freiburg, Faculty of Medicine, University of Freiburg, Freiburg, Germany
[2] Department of Neurology and Clinical Neuroscience, Medical Center – University of Freiburg, Faculty of Medicine, University of Freiburg, Freiburg, Germany
[3] Department of Neuroradiology, Medical Center – University of Freiburg, Faculty of Medicine, University of Freiburg, Freiburg, Germany
[4] Department of Diagnostic and Interventional Radiology, Medical Center – University of Freiburg, Faculty of Medicine, University of Freiburg, Freiburg, Germany
[5] Medical Physics, Department of Radiology, Medical Center – University of Freiburg, Faculty of Medicine, University of Freiburg, Germany
[6] Fraunhofer Institute for Laser Technology (ILT), Aachen, Germany
[7] Parkinson-Klinik Ortenau, Wolfach, Germany
[8] Center for Deep Brain Stimulation, University of Freiburg, Germany

§ Equally contributing

Supplementary Material

Supplementary Table 1: Stimulation Parameters

	**mean ± SD (range)**
Stimulation amplitude L (mA)	2.50 ± 0.70 (1.2-3.8)
Stimulation amplitude R (mA)	2.62 ± 0.56 (1.6-3.8)
Stimulation pulse width L (µs)	59.57 ± 2.09 (50-60)
Stimulation pulse width R (µs)	60.00 ± 0 (60-60)
Stimulation frequency L (Hz)	126.96 ± 14.60 (60-130)
Stimulation frequency R (Hz)	126.96 ± 14.60 (60-130)

Abbreviations: SD, standard deviation

Supplementary Table 2: Euclidean distances from centers of VAT to established DBS sweet spots

	**mean ± SD (range)**
Mean Distance (L/R) to Sweet Spot Dembek (mm)	1.88 ± 0.53 (0.99-2.99)
Mean Distance (L/R) to Sweet Spot Horn/Caire (mm)	1.89 ± 0.61 (0.77-3.22)
Mean Distance (L/R) to Sweet Spot Bot (mm)	1.79 ± 0.62 (0.88-3.09)
Mean Distance (L/R) to Sweet Spot Akram (mm)	3.05 ± 0.89 (1.07-4.97)
Distance to Sweet Spot Dembek contralateral to clinically more affected side (mm)	2.08 ± 0.82 (0.68-3.60)
Distance to Sweet Spot Horn/Caire contralateral to clinically more affected side (mm)	2.10 ± 0.87 (0.57-3.73)
Distance to Sweet Spot Bot contralateral to clinically more affected side (mm)	2.01 ± 0.89 (0.65-4.10)
Distance to Sweet Spot Akram contralateral to clinically more affected side (mm)	3.24 ± 1.21 (1.26-5.59)

Abbreviations: SD, standard deviation
